# Evidence-based patient choice: a prostate cancer decision aid in plain language

**DOI:** 10.1186/1472-6947-5-16

**Published:** 2005-06-20

**Authors:** Margaret Holmes-Rovner, Sue Stableford, Angela Fagerlin, John T Wei, Rodney L Dunn, Janet Ohene-Frempong, Karen Kelly-Blake, David R Rovner

**Affiliations:** 1 Department of Medicine, C214 East Fee, Michigan State University, East Lansing, MI, USA; 2 The Clear Language Group, Biddeford, ME, USA; 3 Department of Urology, University of Michigan, Ann Arbor, MI, USA; 4 VA HSR&D Centre for Practice Management and Outcomes Research and Department of Internal Medicine, Veterans Affairs Hospital, Ann Arbor, MI, USA

## Abstract

**Background:**

Decision aids (DA) to assist patients in evaluating treatment options and sharing in decision making have proliferated in recent years. Most require high literacy and do not use plain language principles. We describe one of the first attempts to design a decision aid using principles from reading research and document design. The plain language DA prototype addressed treatment decisions for localized prostate cancer. Evaluation assessed impact on knowledge, decisions, and discussions with doctors in men newly diagnosed with prostate cancer.

**Methods:**

Document development steps included preparing an evidence-based DA in standard medical parlance, iteratively translating it to emphasize shared decision making and plain language in three formats (booklet, Internet, and audio-tape). Scientific review of medical content was integrated with expert health literacy review of document structure and design. Formative evaluation methods included focus groups (n = 4) and survey of a new sample of men newly diagnosed with prostate cancer (n = 60), compared with historical controls (n = 184).

**Results:**

A transparent description of the development process and design elements is reported. Formative evaluation among newly diagnosed prostate cancer patients found the DA to be clear and useful in reaching a decision. Newly diagnosed patients reported more discussions with doctors about treatment options, and showed increases in knowledge of side effects of radiation therapy.

**Conclusion:**

The plain language DA presenting medical evidence in text and numerical formats appears acceptable and useful in decision-making about localized prostate cancer treatment. Further testing should evaluate the impact of all three media on decisions made and quality of life in the survivorship period, especially among very low literacy men.

## Background

Patients are increasingly urged to become involved in treatment choices, including watchful waiting (surveillance but no active treatment), by working through medical evidence to decide on a course of action with their physicians. Guidelines and evidence summaries are commonly available to physicians and frequently include a "summary for patients", as do some major clinical journals. Decision aids have proliferated in recent years yet most require high levels of literacy to access them and have not been designed according to the principles of plain language. This paper describes one of the first attempts to make a decision aid accessible to consumers with lower levels of literacy and education using principles from reading research and document design. To develop a prototype plain language decision aid, and to describe the design process, we designed a decision aid for treatment decision making in localized prostate cancer. Three standard therapies, surgery, radiation therapy, and watchful waiting, are available to patients and paid for by most insurers in the US. The 10-year mortality rate for each of the standard treatments is equivalent, but the side effects and the processes are quite different. This conundrum is made even more difficult because specialty physicians tend to recommend treatments performed by their own specialty [1,2]. Even inter-disciplinary case conferences can expose patients to evidence and professional opinions that appear to conflict.

The aim of the research reported here was to develop a plain language decision aid for localized prostate cancer initial management following a positive biopsy. Three decision aids (booklet, Internet, and audio-tape formats) were designed to contain equivalent information. Formative evaluation was designed to test acceptability to men with both highly functional and limited literacy skills. We wanted to make sure that the DAs were not perceived as "dumbed down" either in tone or content. A further aim was to identify missing information, and information that might not adequately represent the cancer experience, The aim was to include the perspective of community dwelling men in the appropriate age group and men who had had treatment for their prostate cancer.

### Evidence-based patient choice

"Evidence-based patient choice" is a term coined by Hope in 1996 and elaborated in a book by the same title [3] to articulate the addition of the patient perspective to the evidence based paradigm laid out by Sackett [4]. Adding "patient choice" to evidence-based medicine is an effort to locate the need for evidence squarely in a patient-centred approach to patient-provider encounters. The theoretical roots are in economics, ethics, psychology and medicine. There are also political influences, including the consumer choice movement and struggles over resource allocation and health care organization. The synthesis of ethical approaches to shared decision making, and rational choice models provoke both theoretical and political concerns, and is far from a perfect resolution. The practical task of supporting patients' access to information they can use led us to develop this new prototype. We also desired to have the information available in real time to reach the most satisfying resolution of treatment dilemmas. By making our assumptions transparent, and testing the results with patients and scholars we hoped to improve both theory and practice.

### Approaches to evidence tools for patients

Decision aids exist in modest numbers, specific to particular clinical conditions. A recent meta-analysis identified 131 different decision aids across a variety of clinical problems. Sixty-five of these fit the Cochrane criteria and were evaluated in a randomized trial [5]. Decision aids have expanded the scope of traditional patient education materials and draw on several perspectives. These may be classified conceptually as 1) traditional medical, 2) technology assessment (TA), and 3) patient-centred. Traditional patient education materials mirror the medical model didactic elements. They include descriptions of: a) the disease, b) its epidemiology, c) the morbidity and mortality outcomes of the disease in the population, and d) screening and treatment recommendations for either public information campaigns or clinical encounters. This model has been criticized for its emphasis on diseases, rather than on patients and their concerns, and for being paternalistic [6]. Decision aids have addressed the limitations of the traditional medical approach by integrating evidence-based medicine with patient choice and by encouraging shared decision making. It has largely accomplished this with a technology assessment (TA) approach. TA focuses on the quality of the medical evidence, levels of rigor of evidence and providing symmetry in describing risks and benefits of all patient options. The most widely available examples of TA-based decision aids use the Ottawa Decision Support Framework and BMJ Best Treatments approach. The Ottawa Framework asks the decision-maker to choose among treatment or screening choices demonstrated to be effective [7]. It emphasizes clarifying values for outcomes and emphasizes weighing the pros and cons of alternatives in the choice process to arrive at a tentative choice that the patient discusses with the physician. Consistent with evidenced-based health care, it grades the medical evidence for each alternative according to its quality. The Best Treatments approach likewise grades the evidence, teaches patients about uncertainty in data, but does not consistently provide numerical data for side effects [8]. The TA approach is a major step forward in providing patients with accurate evidence for making comparisons among alternatives, but it may suffer from speaking to the professional health care community better than it speaks to patients. To develop a plain language prototype, we accepted most of the premises of the TA approach, but redesigned the presentation of results, using plain language and document design strategies.

### Translation of evidence into plain language

The rationale for "re-constructing" decision aids in plain language is that it works for patients with a wide range of literacy abilities. Plain language includes three major elements: 1) The use of everyday language and other clear writing strategies, 2) well-structured, logically sequenced, and focused information, 3) effective design and layout. It uses medical evidence as a base and attempts to lead readers through the relevant elements to provide an opportunity for synthesis and drawing conclusions about which therapy best suits the values, fears and expectations of the patient. A plain language approach incorporates social marketing principles and is often used by pharmaceutical companies, patient advocacy groups, and others to deliver compelling messages. The materials these groups produce, however, sometimes minimize or eliminate numbers in the interest of being non-threatening and engaging and accommodating limited quantitative literacy skills[9,10].

Plain language approaches have also been used to communicate public health messages to patients. They frequently attempt to persuade them to undertake an action (e.g., engaging in cancer screening) rather than engage in shared decision making. Highlighted by the recent Institute of Medicine report [11], many groups have published plain language document design guidelines [12-17].

As stated by Hibbard and Peters [18], the challenge is "how to present\ldotsinformation so that it is actually used in decision making." They note that three information presentation strategies assist consumers, all of which are reflected in a plain language approach: lowering the cognitive effort required to use information; linking information to real-life situations to increase the emotional connection; and presenting numbers in frequencies (e.g. 10 in 100). We addressed each of these in developing the prostate cancer DA. To address the immediate world of patients in the midst of being told of a disease and making a decision, it is important to describe the relevant risks and benefits of treatments without losing the precision and realistic risk communication capacity of numbers. Unpacking technical jargon actually adds substantial detail about what will happen to the patient in non-technical, everyday terms. It also explains laboratory tests and other medical information that doctors will use in making recommendations. The purpose is to provide patients with tools and encouragement to engage with the doctor in discussing technical aspects of the information doctors use to make a decision. Along with risk and benefit data, a decision aid should also explain the treatment process to the patient, in order that she or he can anticipate the personal experience of undergoing treatment. This approach does not abandon or delete technical language. Rather it defines it and provides examples, and phonetic pronunciations of medical technical terms in order to improve communication between patients and health professionals.

In the prototype, we introduce the idea that there is variability among patients in their values for potential outcomes. Unlike some decision aids that provide a rational recommendation based on formal utility assessment [19], this approach does not make a recommendation. The resolution of the values dilemma is left to patients, their families and friends, and health care providers.

### Needs assessment

Prior to developing a new decision aid, we performed a two-part needs assessment. In the first part, we searched the literature for surveys providing information about knowledge levels of newly diagnosed patients following biopsy and discussion with their doctors but before treatment. In the second part, we evaluated existing patient education materials for localized prostate cancer. Since we found no surveys of men actually facing the localized prostate cancer treatment decision, we interviewed 184 men newly diagnosed with localized prostate cancer from both community and tertiary care urology practices in Michigan [20]. This sample served as the historical controls for this paper. The survey included demographics, patient knowledge about their prostate cancer, treatment options, side effects and satisfaction with care. Bivariate and multivariable analyses were performed to adjust for potential confounding and to determine if sociodemographic factors affected understanding of key issues. Average age of the men was 64 years and 19% were African American. Seventy-one percent completed some college and 51% of patients were currently employed. White men reported higher rates of knowing their PSA, stage and grade (92%, 93% and 99% respectively) than African American men (69%, 88% and 85%). Ninety-three percent of all respondents stated that they had discussed treatment options with a physician. Only 62% knew that radical prostatectomy, external beam radiation therapy, brachytherapy and watchful waiting were standard options for localized prostate cancer. Significant associations between sociodemographic factors and a patient's understanding of prostate cancer treatment issues were identified. Despite objective gaps in knowledge, men showed high satisfaction with information received from their physicians.

Prostate cancer has been the subject of several decision aids [21,22] Considerable research has identified patient and health professional concerns about treatment. In part 2 of the needs assessment, we used our previously developed generic evidence template to provide evaluation guidelines, but made it specific to prostate cancer [23]. We used the template to comprehensively survey patient education materials publicly available from US government agencies, charities, drug companies and others for inclusion or absence of the evidence elements. The full report found key evidence to be missing from most patient education materials (PEMs) [24]. We found that standard treatment options were almost never explicitly compared to one other. PEMs frequently did not describe the chances of dying from prostate cancer or of experiencing other side effects of each treatment. We found most did not reflect consideration of the user in the way materials were written, structured or designed. Finally, most did not encourage patients to formulate a treatment preference to discuss with their doctors.

Based on the needs assessment, we developed the decision aid, described in this report, in three formats (booklet, audio tape, and Internet) with particular attention to plain language and to potential concerns of African American men. Three major challenges lay at the heart of the plain language translation: 1) introducing the social and emotional context, 2) translating the outcome and side effect rates into plain language, and 3) encouraging shared decision making. The three formats used the same words and content organization to facilitate evaluation of the impact of print, audio and Internet media on patient knowledge and patients' assessment of the usefulness of the different media in preparing to talk with the doctor.

## Methods

### The decision aid prototype: design method and assumptions

Based upon our earlier analysis of good and bad aspects of the prostate cancer PEMs and an extensive search of the clinical literature, a draft of the decision aid was prepared in what might be called the standard medical parlance. This material was iteratively modified to emphasize shared decision making and incorporate the principles of plain language. The research team who designed the project was strongly guided at this point by two health literacy/plain language experts. Similarly, document design and web design were strongly guided by graphics experts. The prototype was presented to the Michigan Prostate Cancer Action Committee (PCAC), a sub-committee of the Michigan Cancer Consortium (MCC), a consortium of hospitals, universities and cancer centrss in the State of Michigan. Final edits were made based on the formative evaluation by patients, clinicians, and the development collaborators and the final document produced.

The prototype booklet and Internet version that resulted from the evaluation process can be viewed at the Michigan Cancer Consortium (MCC) website [25]. Table 1 lists the design decisions made for the prostate cancer prototype.

**Table 1:** Information and Information Architecture Issues and Solutions

**Concern**	**Solution**
Complex information	• Organize and structure document for ease of understanding and use, including use of summary tables
	• Create affective appeal using individual vignettes
	• Describe all elements of treatment process patients experience

Engage the reader in the social context	• Use pictures captioned with comments from men of varying ethnicities
	• Suggest connection with trusted others and other patients
	• Recognize and address feelings of fear

No mandatory autonomy	• Suggest that decisions may be made by the patient or delegated to the doctor, or trusted individual.
	• Suggest shared decision making is compatible with keeping or delegating

Anticipate cultural issues	• African Americans are likely to be sensitive to potential to withhold treatment in the name of watchful waiting.
	• African Americans frequently are diagnosed with more severe disease relative to whites.
	• Sources of authoritative advice vary among ethnic groups. These can be acknowledged subtly in text

Translate medical words, but do not eliminate technical information	• Define words and include a glossary
Creating/choosing appropriate pictures	• Simplify anatomical drawings to include only necessary visual information
	• Include realistic picture of torso, with head included to provide conceptual context
	• Include photographs of men who look serious, but not devastated

Graphic Design and Layout: Use evidence-based document design principles	• Spacing to emphasize different points and topic changes
	• Wide margins to make text less dense
	• Adequate page size to keep information on a topic on one page
	• Graphic design that draws the reader through the booklet
	• Text design that keeps related information in close proximity

Plain language	• Use conversational words and sentence structure, even if not standard grammar
	• Keep the writing tight. Eliminate unnecessary words.
	• Keep the tone friendly and personal, speaking directly to the reader.
	• Explain technical language even though it adds words. Assumptions behind technical words must be included
	• Make scientific references available on the website, but do not include in the text

### Connecting to patients' social and emotional world

To connect with the concerns of men and their families and friends about prostate cancer treatment, the decision aids embed a number of emotional "hooks", showing photographs of middle-aged men of varying races. Quotes from different men state varying preferences for their preferred degree of autonomy: making the decision themselves, delegating it to family or the doctor, sharing it with clergy, friends, or health professionals. These statements are designed to legitimize a range of appropriate alternatives for the problem of "who decides". They are meant to avoid a "mandatory autonomy" position, allowing the patient to hand off the decision to the physician if he does not want to decide himself [26]. The central message promotes active patient participation, followed by making, sharing, or delegating the decision. Further, an attempt is made to imbed in the text items that speak to culture-specific issues. For example, the statement is made that watchful waiting does not mean that the doctor "just does not want to treat me." This statement anticipates the common belief, particularly among African American patients, that doctors may withhold treatment for cost or other reasons.

The emotional context of the booklet is also invoked by explicitly noting in the text that cancer is scary, and that treatment side effects may have heavy emotional impact. The emotional "threat" level of how the disease is discussed is planned to be at a moderate level. The decision aid acknowledges inevitable fear, supplies concrete understanding of disease and treatment, and encourages a sense that patients are competent to make the decision.

### Presenting quantitative evidence

The goal of an evidence-based decision aid is to make the information easy to read, remember, and use. The prostate cancer prototypes use specific textual, organizational, and design elements enhanced by appropriate message framing. They do not assume the reader knows either the technical meaning of words or the background context in which they are used. This filling in of the "why" and "what for" of laboratory tests, treatment regimens, and other medical information is essential for the millions of adults with limited literacy skills and limited experience with navigating the medical system. It is also preferred by most people when learning new information, especially in a highly charged emotional context. Specific guidelines for the prostate cancer decision aids were: 1) aim for low to average reading level (grade 7 level), 2) present quantitative risk information as simply as possible, both positively and negatively framed [27], and 3) use a minimum of technical medical terms, and provide a glossary, called "explanation of medical terms". The objective is to invite all patients to grapple with the decisions they face with as little intervening struggle with technical medical language as possible. The result, ideally, is sophisticated ideas in plain language.

To invite the patient into the content requires attention to "information architecture" and the layout of information on pages to guide the reader through the text. Thus, the density of information on a page, the choice of a booklet or an electronic tool, and the length and "feel" of the information tool become important. The organizing principle was to describe each treatment option in a complete synopsis on its own on two facing pages of the booklet, or on sequential web pages under one button. The audiotape is organized in "chapters", like a "book on tape". For each treatment, the text describes, "What happens", "How this treatment can help", and "How this treatment may cause problems." Each of the individual synopses tells the whole story about a specific treatment. The treatment options are followed by two comparison tables, one narrative and one quantitative. The summary tables explicitly compare results across treatments. Very brief directions guide the reader in how to use the tables. Additional information, not related explicitly to the choice (stage and grade of cancer, and PSA tests), is described in the same amount of detail as a single treatment option (two facing booklet pages). The objective is to demystify what will happen, show how doctors decide what is best, describe the degree of uncertainty about test outcomes, and indicate the medical information on which decisions are based.

Side effects and mortality are presented as mean values, not including the ranges. This is a pragmatic solution to the simplification task, and should be tested further. Explaining that diagnostic test results are imperfect information, and that treatments have only a probability of success is a new idea to most patients. The design task was to present uncertainty, but keep it simple. Another quantitative principle that is critical to informed consent is presentation of absolute, rather than relative risks in terms of specifying a number out of 100 rather than a percentage. Absolute rates have been demonstrated to provide patients with improved estimates of their own chances of experiencing a particular outcome [28].

### Shared decision making

A third challenge was to encourage shared decision making without specifying the form of the clinician-patient conversation or the values discussion. The message on the cover of the DA first introduces the concept of choice, that "there is no right answer" to this treatment problem. The general statement, "Your treatment decision is a shared one between you and your doctor" is later stated directly. A "things to think about" section at the end of the booklet asks a patient to identify his most important goal for treatment, such as curing cancer, ameliorating symptoms, having the best possible sexual performance, or good bowel and bladder control. Patients are also asked to write down the best and worst thing for them about each treatment option. These sections are designed to facilitate an open discussion with the physician without directing it.

The booklet was provided to each person with a request to provide written or verbal feedback. The booklet form of the decision aid was finished first, and provided the text and order of presentation that were duplicated in the audiotape and Internet versions. Qualitative methods were used to evaluate and revise the booklet before survey testing was performed with a new sample of men newly diagnosed with prostate cancer.

## Results

### Formative evaluation

Evaluation was performed in two phases. Phase 1 was a focus group evaluation of the draft language. Phase 2 was a survey follow-up of 60 men newly diagnosed with prostate cancer, using the DA in real-time decision making. The Michigan State University and University of Michigan human subjects IRBs approved all study materials and procedures. Verbal and written consent for participation was obtained at the beginning of interviews and patient encounters.

### Phase 1: focus groups

#### Design and procedure

Focus group methods were used to identify unacceptable or confusing language and to identify missing information or information needed to fully represent the important issues for decision making. This information was obtained from the perspective of men who had not experienced prostate cancer, and also those who had. The analytic techniques used were similar to ethnographic techniques, although cross-sectional rather than longitudinal qualitative reports were used [29].

Homogeneous groups by race were formed to encourage participants to feel comfortable and to express concerns about bias. A moderator's guide addressed the acceptability of content and language, perceived understandability, and usability of the format. We inquired specifically about text passages that described risk numbers, results of a randomized clinical trial, the meaning of laboratory tests and their implications for mortality and treatment. Open-ended questions asked men their perception of the DA main message.

Four focus groups were conducted. Each session lasted between 1.5 and 2 hours and was facilitated by a physician. Booklets were mailed to participants two weeks prior to the focus group and participants were encouraged to review the booklet in detail to provide feedback during the focus group session. Confidentiality ground rules were established and agreed upon by participants. After the session, a twenty-five dollar honorarium was provided to compensate participants for time and travel. Immediately following participants' departure, a debriefing session was held between the facilitator and assistant to develop and amend observation notes and record suggestions for subsequent focus groups. All focus groups were tape-recorded.

#### Sample

Post-treatment men who had undergone surgery or radiation therapy for prostate cancer were recruited from a tertiary care prostate cancer clinic. Community men at risk for prostate cancer due to age were recruited from a study of decision making in benign prostatic hyperplasia. Actual patients at the point of decision making were not included in the focus group, due to their potential vulnerability to inaccurate, misleading, or offensive content. Twenty-five men participated in four focus groups. Mean age was 64.5 years (range 49–80). Twenty-four had health insurance; approximately half were not college educated.

#### Focus group results

The men found the prototype booklet "encouraging" and "comforting." The general consensus was that prostate cancer appeared to be something "that can be dealt with." The DA was described as "professional," the "right size" (8 1/2 × 11), and containing the right balance between the size of white space and of print. The summary table of treatments and risks and the comparison table were viewed as especially helpful and men stated that these clarified individual treatment descriptions. The level of detail was described as being "just right", with the exception that the decision aid did not include detailed description of non-standard treatments (cryosurgery and nerve-sparing surgery). These were added, as was a recommended diagram showing cancer spreading to the surrounding lymph nodes and nerves.

#### Positive and negative framing of statistical information

The booklet stated each side effect as both a number out of 100 chance of occurrence and that number subtracted from 100 as the chance of avoiding occurrence. However, the obvious redundancy was interpreted by some readers as talking down to them and insulting their intelligence. To make it less repetitive, the wording was changed to vary the outcome named and acknowledge that this was another way of stating the outcome. Table 2 shows major changes made based on feedback.

**Table 2:** Booklet Improvements based on formative evaluation

**Information item identified in focus groups and approved by investigators**	**Draft 1**	**Draft 2**
Emotional impact	None	Hearing that you have prostate cancer may shock or frighten you, your family, and your friends. These feelings are natural. They may change over time, as you learn about your diagnosis, make treatment decisions, deal with symptoms, and go on with your life. Men are often afraid to share their feelings or get help from a counselor if needed. If strong feelings are hurting you or your family, ask your doctor to suggest help.
Positive and negative framing	"If 45 men out of 100 experience impotence, this means that 55 do not."	"...about 45 men out of 100 have permanent impotence. This means that 55 men out of 100 will have their original level of sexual activity."
African American differences	None	"African American men are often diagnosed at a younger age than white men and with more advanced prostate cancer. However, treatment may be equally successful for both groups if given the same care."
Specific drug name,	"like Viagra"	"medicine that helps with erections"
Treatment detail	No mention of cryosurgery or nerve-sparing surgery	Included under "newer treatments".
Drawing	Three inset pictures	Only two
Watchful Waiting description	Few disadvantages listed	Added disadvantages regarding potential progression of disease

#### Perspectives of African American men vs. White men

While all found the booklet professional and a good source of information, there were differences in what issues the groups discussed. For African American men, the multicultural photos were important in that they illustrated that prostate cancer affects men of any race. The participants did suggest inclusion of more specific mortality information by race. (See Table 2).

African American men discussed several aspects of fear, including fear about acknowledging health problems, and the absence of fear in the faces of the men in the photos. They characterized the overall message of the booklet as not to be afraid. They also cited fear and distrust as major obstacles preventing their acknowledging a health problem. African American men did not feel the text talked down to them and felt the words were manageable – "there are no big words in this booklet." One commented that, "I didn't feel like you were treating me like a 3^rd ^grader." Lastly, the African American men read the overall message of the booklet as one of "don't be afraid," whereas the White men read the message as one of "informed decision making". Several men across groups commented that they rarely read health related materials, but that they read every word before coming to the focus group. "I normally only read sports. But I read this straight through".

#### Post-treatment men vs. community men

Men who had been treated for their prostate cancer appeared to find discussing the DA more difficult than did the men of the same age who did not have prostate cancer. While they appreciated the information, some indicated that they were not convinced that they really had a choice when deciding on actual therapy, either because of the doctor's presentation or their own concerns about cancer. They also indicated that the consequences of the treatment decision were more emotionally difficult than they anticipated. These men appeared to struggle with the emotional and psychological consequences of their own treatment decisions. Post-treatment White men suggested that the emotional impact of treatment be more strongly addressed as illustrated by the following comments:

"But this doesn't deal with... your first issue is to stay alive and the second issue is the quality of your life afterwards and that may be whether you are incontinent or maybe whether you don't have erections.....But to the fact that, uh, you need to talk to your surgeon about the psychological consequences of the expected outcome of this surgery. Maybe you only think about living first, but looking at it in retrospect, I was like.... Whoa, why didn't you tell me that ahead of time? Not that I would have made a different decision. I just want to be well informed. I didn't get that".

Some post-treatment men felt that the booklet was biased toward watchful waiting as a treatment choice. This was addressed by adding specific negative aspects to watchful waiting in the summary.

For example:

"But this book, uh, had in mind a definite cast toward watchful waiting as opposed to more active interventions and I agree that, uh, that in fact for many of us that was not a very viable option I mean intellectually or emotionally or whatever" and "But as I said before, I thought there was a subliminal message there that said that watchful waiting was probably a preferred choice."

### Phase 2: survey

To test the usefulness of the revised decision aid to men at the point of decision, a new convenience sample of 60 men newly diagnosed with prostate cancer on the basis of biopsy was recruited to test the usefulness and influence of the DA in real-time decision making. Participants were recruited and provided a decision aid after receiving their prostate cancer diagnosis at a post-biopsy visit to their urologists. The urologist introduced a research assistant to patients at the end of the visit in which he was told of his cancer diagnosis. The patient was asked if they would like to be in a research study evaluating the effectiveness of the three different decision tool formats. Patients were recruited sequentially until 20 had been accrued to use the booklet. After the Internet and audiotapes were developed, 40 additional patients were recruited to evaluate these formats. Due to the necessity for computer access for use of the Internet version, these men were offered a choice of the audiotape or Internet decision aid until 20 men were recruited for Internet and 20 for audio-tape use. A telephone survey lasting approximately 20 minutes was conducted by trained survey researchers from the Institute for Public Policy and Social Research of Michigan State University.

The survey included knowledge items taken from the previous needs assessment. DA evaluation questions assessing balance, clarity, and length of the DA were modeled after those of Barry et. al. [30]. Demographic (age, race, income, education and geography) and disease (prostate specific antigen (PSA), cancer stage, and grade) characteristics were ascertained during the interview. Knowledge questions included knowing one's own test results (PSA, stage, grade), treatment options and side effects.

#### Sample

The sample of 60 men recently diagnosed with prostate cancer had an average age of 62 years (SD = 7.9). Ninety percent reported their race as White (8% as African American), 5% had not graduated from high school, 20% had a high school dipoloma or GED, 32% had some college training, 19% had received a bachelors degree and 24% had received training post-baccalaureate. Sixty percent were employed, 33% were retired, and 5% were unable to work. Twenty-two percent had an income of less than $25,000 and 77% reported Internet access.

#### Survey results

The responses of men using the three different media (internet, audio, booklet) were virtually identical across all survey items with the exception that those receiving the audio version were less likely to share it with family and friends (data not shown). Table 3 shows that the sample of men utilizing the DA and historical controls had similar knowledge of their own pathology results, with the exception that the DA sample were less likely to be informed of the stage of their cancer (Fisher's Exact Test P-value = 0.003). Knowledge of treatment options was not significantly different between the two groups. However, 12% more men believed watchful waiting to be a standard treatment in the DA group (87% vs 75% in controls, p = 0.073). In addition, 6% fewer men believed external beam radiation to be a standard treatment in the DA group (88% vs 94% in controls, p = 0.159).

**Table 3:** Knowledge: DA sample vs. historical controls

	**DA sample (N = 60)**	**Historical controls (No./Total)**	**Fisher's Exact Test P-value**
**Knowledge of personal pathology results**			
Knew PSA	56 (93%)	160/181 (88%)	0.34
Informed of stage of cancer	53 (88%)	178/181 (98%)	0.00
Knew grade of cancer	57 (95%)	178/184 (97%)	0.69
**Knowledge of treatment options**			
Knew all of age, grade, stage, health, and PSA are at least somewhat important for treatment decision.	52 (87%)	142/184 (77%)	0.14
Believed watchful waiting to be a standard treatment	52 (87%)	138/184 (75%)	0.07
Believed external radiation to be a standard treatment	53 (88%)	173/184 (94%)	0.16
Believed brachytherapy to be a standard treatment	47 (78%)	143/184 (78%)	>0.99
Believed surgery to be a standard treatment	57 (95%)	177/184 (96%)	0.71
Discussed watchful waiting with physician	28 (47%)	72/184 (39%)	0.36
Discussed external radiation with physician	53 (88%)	166/184 (90%)	0.63
Discussed brachytherapy with physician	38 (63%)	121/184 (66%)	0.76
Discussed surgery with physician	59 (98%)	163/184 (89%)	0.02
**Knowledge of side effects**			
Knew surgery associated with incontinence	59 (98%)	172/184 (93%)	0.20
Knew surgery associated with impotence	58 (97%)	172/184 (93%)	0.53
Knew surgery associated with painful bowel movements	33 (55%)	82/184 (45%)	0.18
Knew radiation associated with incontinence	48 (80%)	113/184 (61%)	0.01
Knew radiation associated with impotence	56 (93%)	145/183 (79%)	0.01
Knew radiation associated with painful bowel movements	46 (77%)	118/184 (64%)	0.08

Discussion of treatment options with the physician showed a significant increase in surgery discussions (98% in DA group, 89% in controls, p = 0.019). Discussions of watchful waiting increased by 6% to 47% in the DA group, and discussions of radiation decreased by 2% (external beam) and 3% (brachytherapy). While not significant, and very small, these changes are consistent with the increase in knowledge of options and side effects.

Table 4 shows that the DA was universally found to be clear and helpful. A substantial percentage of men reported that the DA influenced their decision making.

**Table 4:** Clarity and usefulness of DA (n = 60)

**Amount of information**	Much less than needed 5%	A little less than needed 14%	About right 77%	A little more than needed 4%		DK or refused 7%
**Length**	Much too short 0%	A little too short 11%	About right 84%	A little too long 5%		DK or refused 5%
**Clarity of words**	All clear 44%	Mostly clear 51%	Some clear/ not 5%	Most unclear 0%	All unclear 0%	DK or refused 2%
**Difficulty of numbers**	Very easy 64%	Somewhat Easy 24%	Somewhat difficult 12%	Very difficult 0%		DK or refused 3%
**Treatment description balance**	Complete balance 80%	Slanted to surgery 9%	Slanted to radiation 4%	Slanted to WW 7%		DK or refused 8%
**Recommend DA to a friend?**	Definitely would 78%	Probably would 20%	Unsure 0%	Probably would not 0%	Definitely Would not 2%	DK or refused 2%
**DA improved understanding**	Definitely did 49%	Probably did 42%	Unsure 2%	Probably did not 2%	Definitely did not 5%	DK or refused 2%
**Numbers influenced my decision**	Definitely did 12%	Probably did 41%	Unsure 2%	Probably did not 22%	Definitely did not 22%	DK or refused 3%
**DA helped decision making**	A lot 14%	Quite a bit 30%	Moderate 32%	A little 16%	Not at all 9%	DK or refused 5%

DK = Don't know

Eight-six percent of men reported that they shared the DA with a spouse or partner; 22% shared it with other family members; 14% shared the DA with friends. Seventy-two percent reported that they were more likely to take an active role in their treatment decision.

## Discussion

This attempt to marry evidence-based shared decision making and plain language appears to have been informative and helpful in decision making among a diverse set of men. The design process we used was found to translate medical language while retaining quantitative information. The resulting DA was almost universally found to be helpful in decision making. Men in the DA group were more likely to discuss surgery with their physicians and to know that radiation therapy has side effects. Men reported sharing the DA with family and reported that the DA increased the likelihood of taking an active role in decision-making with the physician. The comparisons reported are limited by the use of historical controls and by the unavailability of data about actual decisions made. However, the two cohorts were surveyed using exactly the same questions. Both cohorts were very well informed about their own test results and about side effects of surgery. Side effects of radiation where less well known and somewhat responsive to the decision aid.

In qualitative analyses, patients identified several principles of the plain language approach as particularly important to them. Those included "translations" of medical language, the emotional difficulties of choices, and the use of what they called attractive layout and illustrations. Some men objected to numbers shown both as a percentage and 100 minus that percentage to rigorously frame each in both positive and negative. However, the provision of numbers to show frequency of side effects of treatments was considered essential. We did not find that patients perceived plain language as "talking down".

It is important to ask what is gained and what is lost in translating evidence-based TA approaches to plain language. Decision aids have been demonstrated to improve knowledge and assist patients in coming to stable decisions earlier in the treatment process [31,32]. In the face of that success, what improvements in decision aid effectiveness may be expected with the plain language integration and what may be lost? The major deletion we have made from the TA approach is the grading of the medical evidence studies used as source material. We presented rates of outcomes but did not indicate whether they came from randomized clinical trials or descriptive studies. Our reasoning is that grading actually distracts from the simple presentation of rates of mortality and side effects of treatments, and that absorbing what the rates mean is the core of what is needed to make an informed choice. References were made available separately.

Health literacy research shows that even highly educated patients who are worried and stressed by a difficult health decision prefer simple, every-day language that is easy to read quickly. No patients asked for references. The credibility of the DA may have been enhanced by their physician's offering it to them and by the endorsement of the Michigan Cancer Consortium (MCC). MCC represents major research universities, the State health department, and 75 other major Michigan medical organizations.

## Conclusion

The plain language DA presenting medical evidence in text and numerical formats appears acceptable and useful in decision-making about localized prostate cancer treatment. The gains lie in the potential to improve on earlier gains in patient knowledge. If the actual content is not diluted, but the language, organization and design are more useful for patients, it is possible that mean knowledge scores may improve and that the median and the mean will converge. Parker et al [9] argue that health literacy is central to multiple health system priorities, including quality, cost containment, safety, and patients' involvement in health care decisions. They suggest that without attention to literacy, the move toward increased patient participation in health care decisions will exacerbate disparities in access and outcomes. This could mean that people with poor health literacy cannot function successfully in an environment designed for active, informed consumers. It should be noted that among our historical controls, there were large and significant deficits in knowledge among African Americans compared with White patients. We did not have sufficient numbers of African Americans in the intervention sample to test the ability of the DA to improve this deficit or to gauge the effect the DA had on willingness to discuss treatment options with physicians. Further testing should evaluate the DA among men with historically low levels or information, as well as among very low literacy men and non-readers. Testing of this DA in a trial against usual care is needed, as is development of the plain language approach in other conditions.

## Competing interests

The author(s) declare that they have no competing interests.

## Authors' contributions

All authors collaborated on conception and design of the study. MHR was responsible for drafting the article, the design of the decision aid evaluation and drafting the template. DRR provided the original expert draft of the DA. MHR, JTW, and AF contributed early changes. SS and JOF drafted the plain language translation. AF and JTW designed, drafted and performed the survey. KKB analyzed focus group data. RLD performed the statistical analyses. All contributed to editing and revising and approved the final manuscript.

## Pre-publication history

The pre-publication history for this paper can be accessed here:


